# New thermoluminescence age estimates for the Nyos maar eruption (Cameroon Volcanic Line)

**DOI:** 10.1371/journal.pone.0178545

**Published:** 2017-05-30

**Authors:** Christoph Schmidt, Jean Pierre Tchouankoue, Peguy Noel Nkouamen Nemzoue, Félicité Ayaba, Siggy Signe Nformidah-Ndah, Emmanuel Nformi Chifu

**Affiliations:** 1 Chair of Geomorphology & BayCEER, University of Bayreuth, Bayreuth, Germany; 2 Department of Earth Sciences, University of Yaounde I, Yaounde, Cameroon; University of Oxford, UNITED KINGDOM

## Abstract

Nyos maar is located in the Cameroon Volcanic Line and generates a multitude of primary and secondary hazards to the local population. For risk assessment and hazard mitigation, the age of the Nyos maar eruption provides some vital information. Since previous dating efforts using a range of techniques resulted in vastly varying eruption ages, we applied thermoluminescence (TL) methods to obtain independent and direct chronological constraints for the time of maar formation. Target minerals were granitic quartz clasts contained in pyroclastic surge deposits. Thermoluminescence plateau results prove that heat and/or pressure during the phreatomagmatic eruption was sufficient to reset the inherited luminescence signal of granitic bedrock quartz. Parallel application of three TL measurement protocols to one of the two samples gave consistent equivalent doses for the quartz ultra-violet emission. Despite the robustness of our dose estimates, the assessment of the dose rate was accompanied by methodological challenges, such as estimation of the original size distribution of quartz grains in the pyroclastic deposits. Considering results from additional laboratory analyses to constrain these uncertainties, we calculate an average maximum TL age of 12.3 ± 1.5 ka for the Nyos maar eruption. Based on these new data, a more solid risk assessment can be envisaged.

## Introduction

Dating of volcanic eruptions in active volcanic environments is of extreme importance in the understanding of possible eruption cycles, geomorphological evolution of volcanogenic landforms and mitigation of natural hazards [1,2]. Classical dating methods for volcanogenic material are based on the decay of radioactive isotopes (e.g., ^40^Ar/^39^Ar dating technique) and tied to specific constraints such as the half-life of ^40^K, which in many cases is too long relative to recent volcanic manifestations that took place only a few thousands of years ago. Further methodological complications comprise the presence of excess argon or the absence of suitable K-rich phenocrysts in the material produced by the eruption. The consequences are often overestimated ages and/or large uncertainty ranges in the order of 20‒50% [3–6]. Surface exposure dating of lava flows using cosmogenic nuclides (e.g., ^3^He or ^10^ Be) was recently proposed but requires complex analytical procedures [7]. Interpretation of ^14^C dates in volcanic contexts is often challenging due to unclear temporal relationship between the dated event (mostly the death of an organism) and the time of eruption [8,9]. Luminescence dating represents a method that can help in systematically dating Holocene and Late Pleistocene eruptions [10,11]. However, glassy volcanic rocks (e.g., rhyolite or basalt) are less suitable for luminescence dating due to so-called anomalous fading [12–14], which relates to signal instability and causes age underestimation. By contrast, the signal carried by volcanogenically heated or fragmented country rock is actually related to the same dated event–the volcanic eruption–but is not affected by fading and therefore offers more reproducible results [15]. Corresponding sampling contexts are for instance bedrock overlain by a lava flow or the tephra ring surrounding a maar diatreme. By specifically targeting xenocrystic quartz crystals from the disintegrated country rock, within volcanic deposits, it is thus possible to determine a direct age of the eruption, provided that heat and/or pressure of volcanic activity completely reset the inherited, geological luminescence signal.

**Fig 1:**
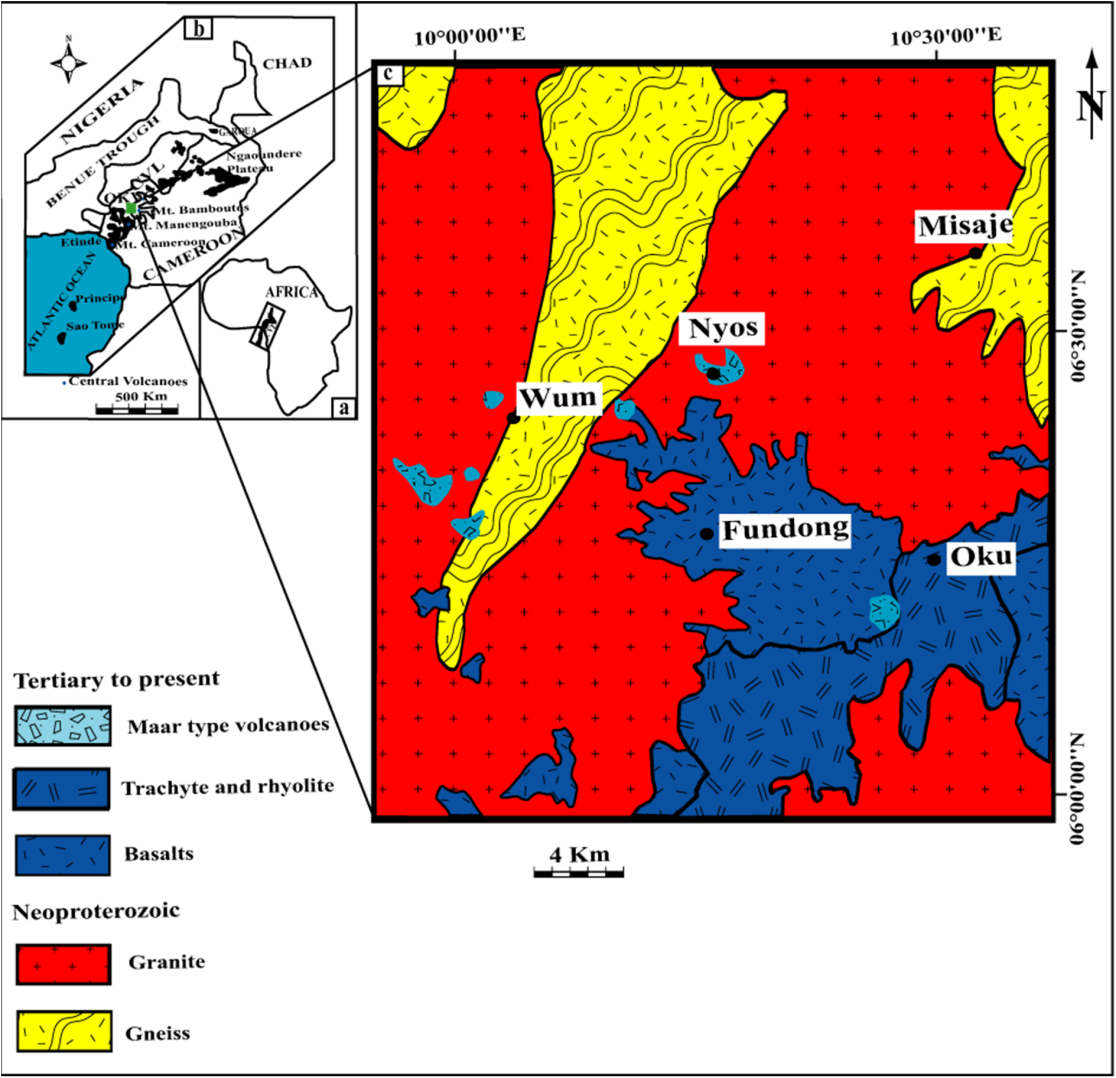
Geological map of the greater study area. (a) Cameroon Volcanic Line in Africa, (b) Oku volcanic field in the Cameroon Volcanic Line, (c) Nyos maar volcano in the Oku volcanic field. Simplified from [17].

**Fig 2:**
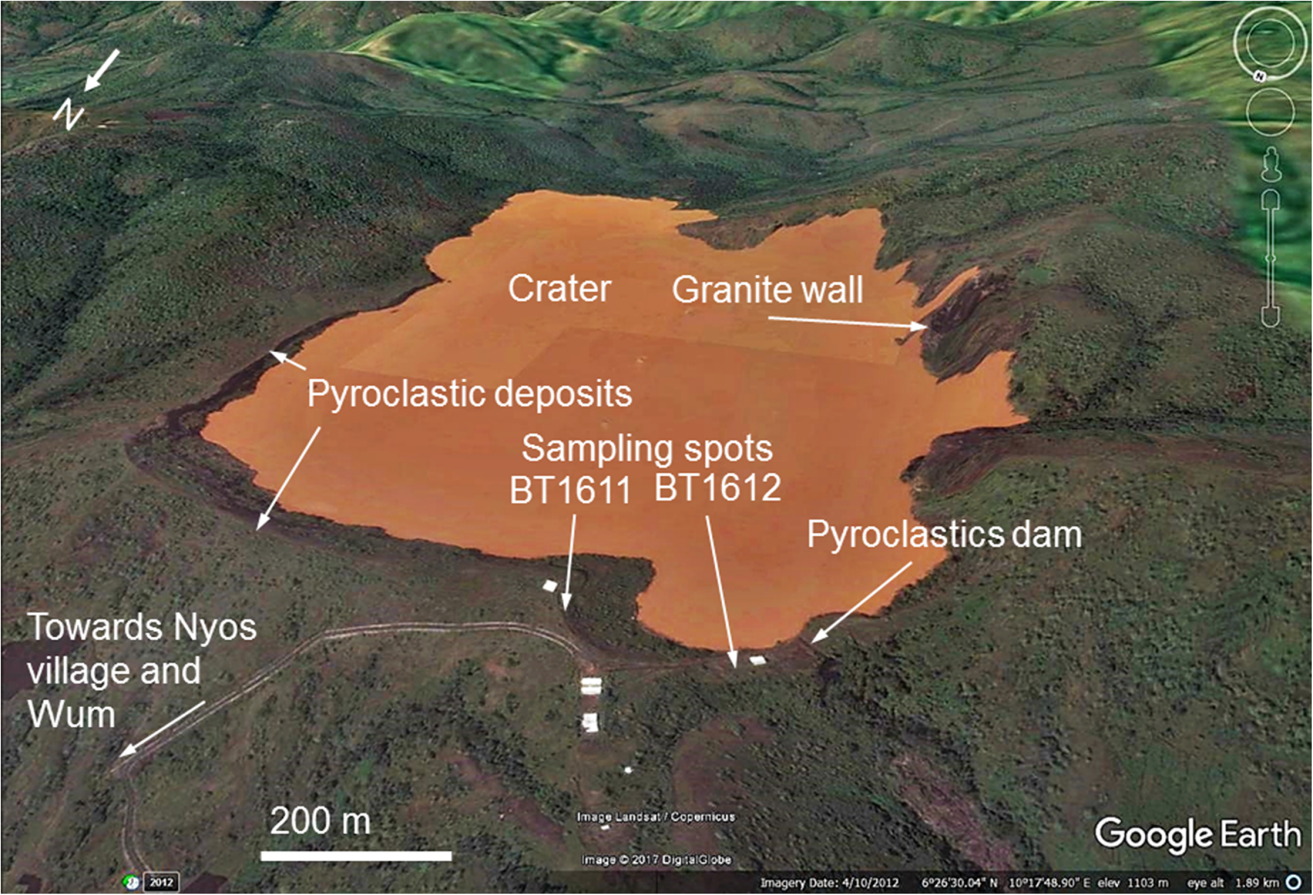
Google Earth satellite image showing the location of luminescence sampling spots.

The Nyos maar, a volcano located in the southern continental part of the Cameroon Volcanic Line (CVL; Fig 1), is of special interest for volcanologists and local policymakers due to its inherent threat to the surrounding population. On 21 August 1986, the release of large amounts of CO_2_ that had accumulated in the lake that occupies Nyos maar killed ~1,700 people and a large number of domestic and wild animals [1]. The main hazard today is a secondary one and connected to a dam made of pyroclastic surge deposits confining Lake Nyos to the north (Fig 2). Collapse of the dam and the subsequent flooding would threaten ~10,000 people living in the valleys below Lake Nyos [16]. For risk assessment in terms of repeated volcanic activity and potential dam failure, knowing the age of the Nyos maar is of paramount importance. Attempts using various dating methods have resulted in ages covering three orders of magnitude (400 a to > 350 ka; see discussion below). In order to better constrain the age of the Nyos maar, we applied thermoluminescence (TL) dating methods to granitic quartz clasts fragmented during the formation of the maar and mixed with volcanic debris. Three different TL measurement protocols and two samples provided consistent and direct age estimates for the Nyos maar eruption, while the optically stimulated luminescence (OSL) signal was found unsuitable as deduced from linear modulation OSL (LM-OSL) experiments.

## Geological background

The CVL (Fig 1A and 1B) is a N30E oriented tectonic structure made up of volcanic islands, continental volcanoes and plutonic and volcanic complexes ranging in age from 82 Ma to present. The CVL bears the active Mt Cameroon with latest eruptions in 1999 and 2000 [18], >100 cinder cones and >40 maars presumed to be not older than 1 Ma.

The Nyos maar (6°27'5''N/10°17'55''E) is located in the southern continental part of the CVL and belongs to the monogenetic Oku volcanic field (Fig 1C) that culminates at Mt Oku (3011 m) (e.g., [8]). Volcanic activity in the Oku volcanic field ranges from effusive to explosive, and a wide range of compositions have been erupted including basanite, basalt, hawaiite, mugearite, trachyte, and rhyolite. Basement rocks are gneisses and granitoids, which formed during the Pan-African orogeny (~600 Ma) [19].

**Fig 3:**
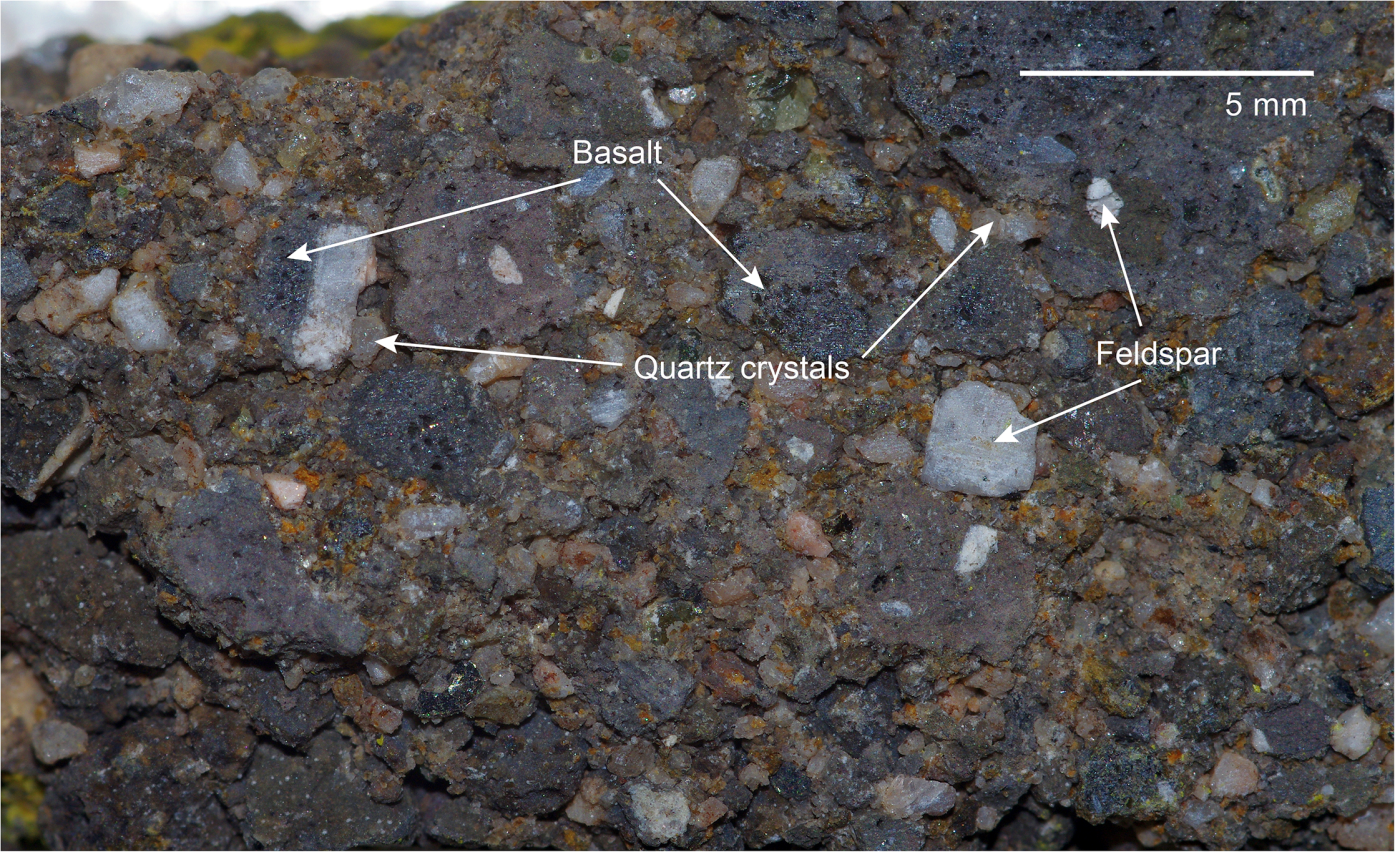
Macroscopic view of the Nyos maar tuff as sampled from the pyroclastic deposits at the crater rim.

The basement of Lake Nyos and its vicinity is mostly formed of granitic rocks of dominant micropegmatitic texture. Basaltic rocks appear as small flows directly overlapping the granitic basement. Pyroclastic surge deposits are found near to the crater and cap the basalt flows to the west of the volcano. Pyroclastic rocks contain broken pieces of basement granites and peridotite xenoliths (Fig 3) [19,20].

## Previous dating of Nyos maar

Various dating methods gave a large age spectrum between 400 a and 350 ka or older for the Nyos maar volcano. The age of 400 a was obtained using the ^14^C dating method [8], while the K/Ar method applied to basalt suggests that the lake is more than 350 ka old [4]. The latter age estimates, however, were deemed unreliable due to inherited radiogenic ^40^Ar [21]. Aerial photography comparison and extrapolation of the 2 m reduction of dam width over the observation period of 34 a resulted in a minimum age of Lake Nyos of 4 ka [4]. Zogning et al. [22] obtained an age of 3.4 ka (^14^C) for the oldest sediments of Lake Njupi, a dam lake located 5 km to the west of Lake Nyos. These authors linked the absence of volcanic debris in sediments of Lake Njupi to the conclusion that Lake Njupi formed following the eruption of the Nyos maar and hence that the eruption of the Nyos maar should be older than 3.4 ka. Radiometric dating based on disequilibria in the U-series yields ages of trachy-basalts/basanites between 3.7 ± 0.5 ka (^226^Ra/^230^Th system) and 8.90 ± 0.52 ka (^226^Ra/Ba–^230^Th/Ba system) for formation of Lake Nyos [23]. However, these ages were not calculated with initial nuclide activity ratios from volcanogenic material sourced from the Nyos maar but with measured values from young basanites at Mt. Cameroon ~250 km to the southwest. Reviewing their results, Aka et al. [23] propose an age between 5 and 10 ka for the Nyos maar.

## Materials and methods

### Sample description and preparation

Crustal xenoliths that can reach 20 cm in size are embedded in volcanic debris that occupy the western flank of the crater of the volcano (Fig 2). The two samples under study (laboratory codes BT1611 and BT1612) were collected on the western side of the maar crater near the pyroclastic dam which frequently overflows during the rainy season (March-October). The sampling points (N6°26’46.43”/ E10°17’41.41”; ~1100 m a.s.l.) are separated by ~200 m distance; the sample closer to the Nyos dam (BT1612) is shown in Fig 3. Collected samples contain a mixture of granite fragmented to various grain sizes embedded in a dark and fine matrix of volcanogenic material. The porphyroid pink granite itself is composed of orthoclase, microcline, quartz, plagioclases and biotite. The target material for luminescence dating were the sand-sized quartz crystals in the dark matrix. Because of their small grain size these are expected to have had the best chance for complete luminescence signal resetting during the maar explosion, either by heat, light, pressure, or a combination of any of these factors.

The two samples were prepared under subdued red-light conditions (640 ± 20 nm) after marking the outer surface with yellow paint to avoid incorporation of light-influenced material into the luminescence sample. The outer layer of at least 2 cm of the brittle samples was removed with a low-speed water-cooled diamond saw; the interior part was then gently crushed in a steel mortar to obtain grain sizes in the range 90‒200 μm. Subsequent chemical preparation included treatment with 10% HCl and 10% H_2_O_2_ to dissolve carbonates and oxidise organic matter, respectively. Heavy minerals and feldspars were removed from the polymineral samples by applying density separation using sodium-polytungstate (2.62 g cm^-3^ < *ρ* < 2.75 g cm^-3^). Etching the remnant grains in 48% HF for 45 min served to remove the outer layer of the grains influenced by external α-radiation and to dissolve any remaining feldspars. Subsequently, samples were washed in 10% HCl to remove fluorides that could have precipitated during etching.

The absence of feldspars in the sample was proven by an IRSL test measurement (*n* > 3 for each sample) that yielded signals at instrumental background level for regeneration doses in the range of the expected equivalent dose (*D*_e_). Regarding the large size of the samples (several 100 g), comparatively few pure quartz grains (~50 mg) were left after sample preparation. Future sampling should take this into account.

### Instrumentation

Luminescence measurements were carried out with a Risø TL/OSL DA-15 reader attached to a DA-20 controller (TL in the UV spectral range) [24] and with a Freiberg Instruments lexsyg standard reader (optically stimulated luminescence, OSL) [25].

The Risø system is equipped with an in-built ^90^Sr/^90^Y β-source delivering a dose rate of 1.97 Gy min^-1^ to coarse grain quartz samples. UVTL signals were detected with an EMI 9235QB UV-sensitive photomultiplier tube after passing through a 7.5 mm Hoya U340 glass filter.

The lexsyg system features an in-built ^90^Sr/^90^Y β-source yielding a quartz coarse grain dose rate of 3.34 Gy min^-1^ and a Hamamatsu H7360-02 photomultiplier tube allowing detection of wavelengths in the range 300‒650 nm. Detection filters further constrained the spectrum to ~330‒380 nm (Delta-BP 365/50 EX plus Schott BG3) for recording LM-OSL. Optical stimulation was achieved by green LEDs emitting at 525 ± 25 nm with a maximum power density of ~36 mW cm^-2^ at sample position.

All TL measurements were conducted in N_2_ atmosphere and using a heating rate of 5 K s^-1^. The background was recorded in a second run on the same aliquot immediately after signal readout and subtracted channel-wise to obtain net signals. Prepared quartz grains were attached to aluminium (Risø) or stainless steel cups (lexsyg) using silicone oil and a 5 mm spray mask to obtain an acceptable signal-to-noise ratio in luminescence measurements.

### Initial tests and measurement protocols

To assess the general suitability of luminescence methods to date the phreatomagmatic eruption having formed the Nyos maar, a range of initial measurements was conducted, specifically aimed at testing

1. whether the eruption mechanism was successful in completely erasing the geological (inherited) luminescence signal of the fragmented granitic bedrock and
2. which luminescence emissions of the quartz samples provide thermally stable and non-fading signals.

To address the first issue, a heating plateau test was carried out for the two samples. A plateau in the ratio of natural TL (NTL) to TL after administering an additive β-dose on top of the natural signal (NTL+ β) demonstrates sufficient luminescence signal resetting during the eruption in this temperature range of the TL glow curve. The heating plateau further indicates the range of a thermally stable luminescence signal for determining the *D*_e_ [26]. The measurements were performed on two aliquots for NTL and two aliquots for NTL+ β for each sample (due to scarcity of sample material). TL glow curves were normalised to the integrated TL signal from 350‒400°C glow curve temperature of a 50 Gy test dose administered after the NTL or NTL+ β signals were measured (second-glow normalisation). This normalisation integral was considered because the heating plateau itself was determined with these measurements; for subsequent analyses the heating plateau range of each sample was adopted.

The optically stimulated luminescence (OSL) signal of quartz can be advantageous over the TL signal because of less severe heat treatments necessary in the course of repeated measurements for *D*_e_ determination, with potentially more reproducible signals. However, a requirement for accurate optical dating is the presence of a so-called fast component in the bulk OSL signal [27]. Therefore, the composition and thermal stability of the OSL signal of both samples was checked by measuring linearly modulated OSL (LM-OSL) [28]. Here, the stimulation power density was ramped from 0 to 36 mW cm^-2^ during a period of 2 h (at 125°C) after bleaching the sample for 60 s in the lexsyg reader using green stimulation (36 mW cm^-2^; 125°C), administering a regenerative β-dose of 500 Gy and preheating at 200°C for 10 s. The resulting LM-OSL curve allows quick visual assessment of presence or absence of the OSL fast component. Furthermore, LM-OSL curve deconvolution provides insights into the relative proportion of fast, medium and slow components [27].

Scarcity of prepared sample material for BT1611 led us to apply a UVTL single-aliquot regenerative dose (SAR) protocol for *D*_e_ determination. The measurement sequence includes four regenerative dose points (giving signal *L*_x_) from which the lowest dose point is repeated to check the accuracy of sensitivity correction (‘recycling ratio’). This was done by means of a constant test dose (giving signal *T*_x_) in between the regeneration cycles. The sensitivity-corrected dose points (*L*_x_/*T*_x_) were fitted with a linear function to deduce a functional relationship between TL and dose, applying the functions ‘calc_TLLxTxRatio’ and ‘plot_GrowthCurve’ contained in the R package ‘Luminescence’ (version 0.6.4) [29–31].

With this protocol, however, a significant sensitivity change of the sample during the first TL readout cannot be detected or corrected for by test dose monitoring. In this case, the SAR protocol would produce systematically inaccurate results. For sample BT1612, we therefore additionally applied a multiple-aliquot additive dose (MAAD) [26] and a single-aliquot regeneration and additive dose (SARA) protocol [32]. While the MAAD protocol is not affected by sensitivity changes in the course of the first measurement, the SARA sequence automatically takes into account these changes. Consistent SAR, MAAD and SARA ages would thus confirm that the SAR protocol yields accurate dose measurements for the investigated samples.

Specifically, for the MAAD measurements the sample material was divided into five portions which were further split into three aliquots. Each portion received different additive β-doses of 0, 20, 40, 60 and 80 Gy prior to TL readout. The integrated TL signal was then plotted against the additive β-dose and the data points fitted with a single saturating exponential function, whose intercept with the negative part of the dose axis gives the absolute value of the *D*_e_. The UVTL instrumental background was recorded separately and its integrated signal subtracted from the bulk integrated signal.

In the course of the SARA protocol, four portions of sample material (three aliquots each) were β-irradiated with increasing additive doses of 0, 20, 40 and 60 Gy. Subsequently, the *D*_e_ was measured by regenerating UVTL signals such that these bracket the signal induced by the natural plus the additive dose. In other words, a SAR protocol without test dose correction was applied. When plotting the obtained (not sensitivity-corrected) *D*_e_ values against the known additive β-dose, the dose axis intercept of the linear fit to these data points returns the ‘true’ *D*_e_ (sensitivity-corrected). The slope of this fit further corresponds to the dose recovery ratio in the SARA experiment, i.e. a slope of 1 suggests that a known added dose was successfully recovered in the measured dose [33].

Finally, a test for anomalous fading was carried out, consisting of repeated cycles of β-irradiation with a dose similar to the *D*_e_ and UVTL measurement (*L*_x_), while a pause of varying duration (up to ~15 h) was inserted before measurement. Test dose cycles in between regeneration cycles provided proxy data for sensitivity changes, and the *L*_x_/*T*_x_ ratio was plotted against the normalised delay to assess signal loss over time (following the approach of Auclair et al. [34]).

### Dose rate assessment

Due to removal of the α-influenced rim of the quartz grains, only external β-, γ- and cosmic radiation contribute significantly to the environmental dose rate. Beta- and γ-dose rates were calculated from K, Th and U concentrations determined from representative bulk maar tuff material (Fig 3) by thick-source α-counting (Th, U) [26,35] and ICP-OES (K). Radioelement concentrations were then translated to dose rates with conversion factors published by Guérin et al. [36] as implemented in the program ADELE [37], which was also used for age calculation.

The size range of quartz grains (90‒200 μm) used for luminescence measurements does certainly not reflect the ‘original’ size spectrum after bedrock disintegration in the course of the maar eruption, which has implications for the amount of β-dose absorption within the grains. The original size distribution of quartz grains was assessed by gently disaggregating representative maar tuff without cracking individual quartz grains followed by density separation to extract quartz only (see section sample preparation). The diameters of several hundred quartz grains were then assigned under the microscope to the size fractions 200‒600 μm, 600‒1500 μm and 1500‒2200 μm, which were weighed subsequently (the number of grains <200 μm and > 2200 μm was negligible). Within uncertainties of our analyses, all three size fractions amounted to almost identical masses, so that we adopted a grain size range of 400‒2000 μm for age calculation. This range certainly covers >90% of the grains studied and should thus adequately account for the effects of internal β-attenuation (see also S4 Fig).

The matrix of pyroclastic deposits contains both fragmented granitic bedrock as well as juvenile volcanic material and hence the dose received by individual quartz grains after the phreatomagmatic eruption is likely to vary from grain to grain. Provided that the dose response of individual grains is in the linear range, the average dose rate determined for the matrix combined with the arithmetic mean *D*_e_ should result in the best estimate for the true burial dose [38]. Potential spatial differences in β-radiation (mm-scale) within the maar tuff and therefore variance in doses received by individual quartz grains are anyway assumed to be averaged out by using comparatively large aliquots (~5 mm in diameter), hosting ~750 ± 200 grains (calculated with the function ‘calc_AliquotSize’ implemented in the R package ‘Luminescence’ [29–31]). Given the resultant *D*_e_ distributions, application of age models such as the Central Age Model (e.g., [39]) does not significantly change the final dose estimate within errors; even the Minimum Dose Model (e.g., [39]) would produce a dose estimate not substantially different from the arithmetic mean (maximal 9% deviation in the worst case scenario). Apart from the insignificant effect, the application of any age model does not appear to be justified considering the complexity of dose rate assessment.

The cosmic dose rate was computed by use of the function ‘calc_CosmicDoseRate’ (implemented in the R package ‘Luminescence’ [29–31]) in combination with topographic information of the samples (longitude, latitude, altitude, overburden). However, the temporal variation of overburden thickness relevant for the luminescence samples is unknown and has to be estimated from geometrical information available for the pyroclastic cone of Nyos maar. Field observations suggest that the initial surface of this cone at the Lake Nyos dam was ~1.5 m higher than today [4]. We therefore approximated the cosmic dose rate by adopting this number for the luminescence sampling sites and assuming constant erosion rate from an initial flat overburden of ~1.7 m to a final cover of ~ 0.2 m using a density of 2.2 g cm^-3^. An assumed error of 10% is thought to represent most of the inaccuracies related to this model.

The saturation water content of representative sample material was determined in the laboratory (~13 wt.%; water weight over dry weight) and 75% of this value was considered to reflect the annual average moisture content in view of the tropical climatic conditions at Nyos maar. Increased error margins of 40% (relative to absolute moisture) should include all conceivable deviations (e.g. topographic effects or long-term change in precipitation), so that we adopted a value of 10 ± 4 wt.% for age calculations. The correction factors given in Aitken [26] served to transform dry dose rates to wet dose rates.

## Results

**Fig 4:**
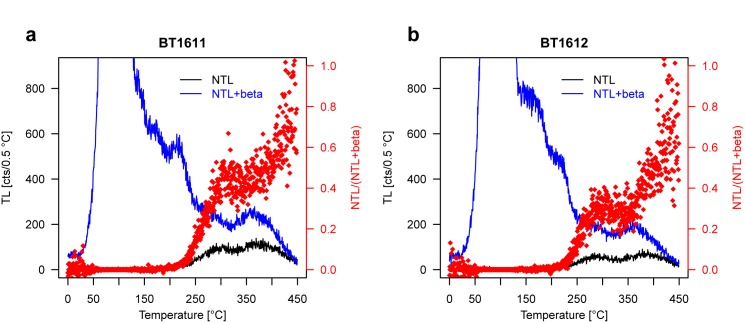
**Heating plateau test for samples BT1611 (a) and BT1612 (b).** The size of the additive β-dose was 25 Gy. The plateau demonstrates luminescence signal resetting in the range ~300‒400 and ~300‒380°C, respectively, and provides integration limits for *D*_e_ determination.

The successful UVTL heating plateau test for both samples proves that the maar eruption completely reset the geological luminescence signal in the plateau ranges 300‒400 and 300‒380°C for samples BT1611 and BT1612, respectively (Fig 4). These are also the integration limits of thermally stable signals for *D*_e_ evaluation, as adopted for the SAR, MAAD and SARA protocols.

LM-OSL measurements and decay curve deconvolution indicate that the signal is–at least for sample BT1612 –not dominated by the OSL fast component but is composed mainly of medium and slow components which are less favourable for dating applications [27] (see also S2 Fig). We therefore relied on the UVTL signal in the heating plateau range for *D*_e_ determination instead.

**Fig 5:**
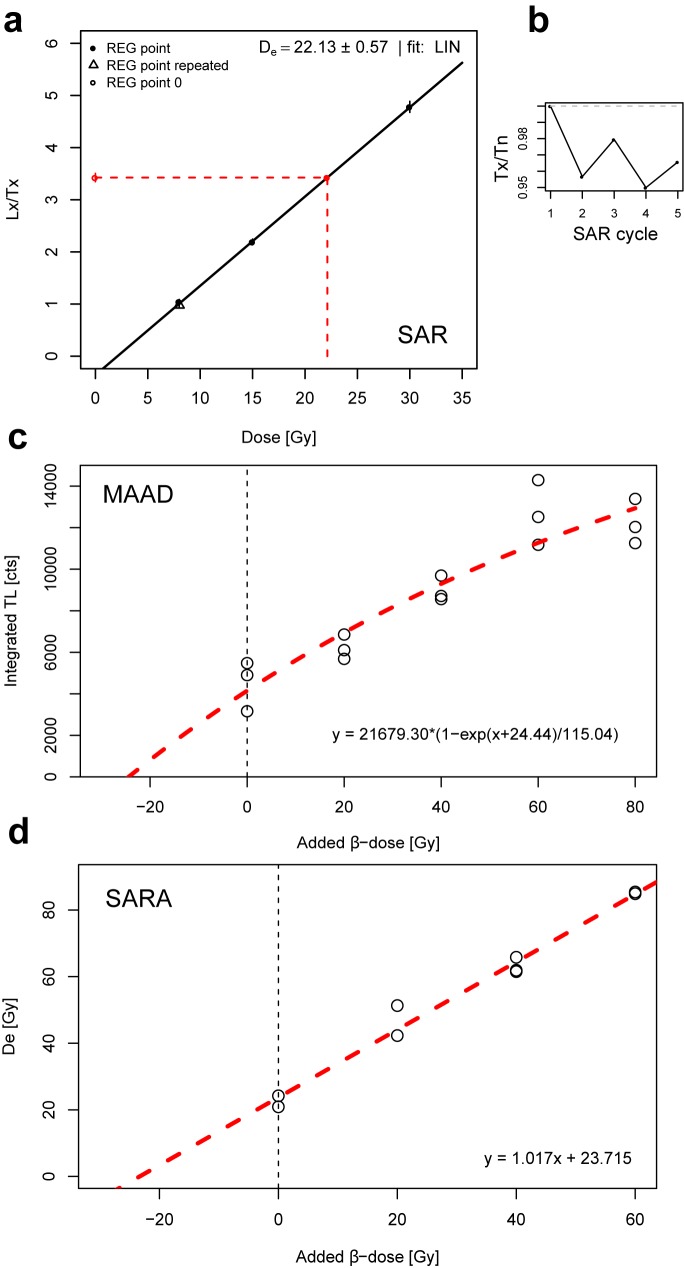
Dose response curves of three methods applied for *D*_e_ determination. (a) Exemplary SAR dose response curve for one aliquot of sample BT1611. (b) Changes in UVTL dose sensitivity in the course of the five SAR cycles, as deduced from the test dose (*T*_x_) measurements. Note that the relative change in sensitivity does not exceed ~5%. (c) MAAD dose response curve for sample BT1612. (d) Plot of UVTL SARA data for sample BT1612. The *D*_e_ values shown on the y-axis were obtained by running a SAR sequence of aliquots having received different additive β-doses, but without correction for sensitivity changes. The known additive β-doses are shown on the x-axis. The linear regression represents on the one hand the degree of sensitivity change during natural TL readout via its slope. On the other hand, it gives the true palaeodose by extrapolating it to the dose axis [32]. Data analysis was performed with several functions of the R package ‘Luminescence’ (v. 0.6.4) [29–31].

**Fig 6:**
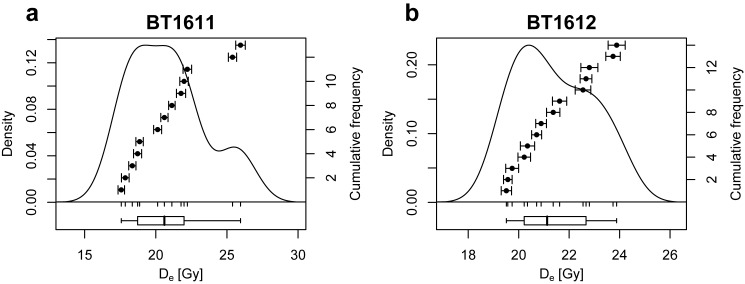
Kernel density estimates of the UVTL *D*_e_ distribution of samples BT1611 and BT1612, measured with the SAR protocol. The overdispersion of these distributions amounts to 12% and 6%, respectively.

Analytical results of radioelement quantification, results of the UVTL SAR, MAAD and SARA measurements, calculated dose rates and TL ages are compiled in Tables 1 and 2. The fact that SAR, MAAD and SARA ages of sample BT1612 are indistinguishable from each other within 1*σ* error margins strongly suggests that the SAR protocol–which is the only method applied for *D*_e_ determination of sample BT1611 –is able to accurately measure radiation doses stored in granitic bedrock quartz from the Lake Nyos area. Representative SAR, MAAD and SARA dose response curves of samples BT1611 and BT1612 are shown in Fig 5. Fig 6 presents the distribution of measured *D*_e_ values of both investigated samples. The overdispersion values of ~12% and ~6% for samples BT1611 and BT1612 are larger than expected given the number of grains per aliquot. Whether this is coupled to incomplete resetting during the eruption or due to dose rate heterogeneity cannot be disentangled.

**Table 1:** Measured radioelement concentrations and calculated wet dose rates. The total dose rate is already corrected for β-attenuation within the quartz grains (using the attenuation factors of [40]) and contains a small contribution of external α-dose rate of 0.002‒0.004 Gy ka^-1^ resulting from the assumed etched layer of 20 ± 5 μm thickness, for which an α-efficiency (*a*-value) of 0.030 ± 0.003 was adopted [41].

Sample	K [wt.%]	Th [μg g^-1^]	U [μg g^-1^]	Water content assumed [wt.%]	*Ḋ*_β_ [Gy ka^-1^]	*Ḋ*_γ_ [Gy ka^-1^]	Cosmic *Ḋ* [Gy ka^-1^]	Total *Ḋ* [Gy ka^-1^]
BT1611	1.17 ± 0.12	6.35 ± 0.76	3.95 ± 0.23	10 ± 4	0.86 ± 0.06	0.92 ± 0.05	0.20 ± 0.02	1.99 ± 0.08
BT1612	1.23 ± 0.12	7.04 ± 0.74	2.33 ± 0.22	10 ± 4	0.78 ± 0.06	0.81 ± 0.05	0.20 ± 0.02	1.79 ± 0.08

**Table 2:** Results of UVTL measurements and calculated TL ages. Recycling ratios are given as average values of all accepted aliquots along with 1*σ* uncertainty range.

Sample	Protocol	*n*^a^	Recycling ratio	*D*_e_^b^ [Gy]	Total *Ḋ* [Gy ka^-1^]	Age [ka]
BT1611	SAR	13/16	0.98 ± 0.05	20.7 ± 3.5	1.99 ± 0.08	10.4 ± 1.8
BT1612	MAAD	‒	‒	24.4 ± 5.2	1.79 ± 0.08	13.7 ± 3.0
	SARA	‒	‒	23.3 ± 3.7	1.79 ± 0.08	13.0 ± 2.2
	SAR	14/15	0.99 ± 0.06	21.2 ± 2.3	1.79 ± 0.08	11.9 ± 1.4

^a^ Number of accepted aliquots in relation to total number of measured aliquots. Acceptance criterion was a recycling ratio in the range 0.9‒1.1.

^b^ The *D*_e_ includes a systematic error of 4% due to β-source calibration. This error was not propagated but added on top once arithmetic mean *D*_e_ values were obtained.

Analysis of the SARA data proved minor sensitivity changes of UVTL during the natural TL measurement in the laboratory, because the regression line in the plot of individually determined *D*_e_ values against the known added β-doses is characterised by a slope of 1.017 (Fig 5C). Therefore, we consider the SARA results as robust.

The fading test carried out on three aliquots of sample BT1612 did not show any signal loss within errors over the observation period (see S3 Fig). However, it must be noted that the sample storage periods prior to TL measurement used in this study (< 15 h) might not be long enough to detect long-term fading.

Based on the 1*σ* confidence intervals, all ages as shown in Table 2 are in agreement with each other and given that they most likely represent one single eruption event, it appears legitimate to calculate an average age for this eruption. The arithmetic mean results in an average TL age of 12.3 ± 1.5 ka, where the uncertainty range was derived from the standard deviation of the four individual ages.

## Discussion

### Quality of luminescence results

The successful UVTL heating plateau test validates that the luminescence signal was reset during the phreatomagmatic maar explosion, which represents a prerequisite for successful dating. This result supports earlier indications that phreatomagmatic eruptions are potentially capable of exerting sufficiently high temperatures and/or pressure to evict all trapped charge from dosimetrically relevant traps in the crystal lattice of fragmented country rock [11,42]. Further evidence that the dated event corresponds to the volcanic eruption is the agreement of TL ages of both samples within 1*σ* error margins. Had other agents of luminescence signal resetting been active in the past (e.g., post-depositional re-location of the volcanic deposits), it would be unlikely for them to cause congruent ages.

The accuracy of applied TL protocols was assured by checking three different approaches of *D*_e_ measurement against each other for one sample, resulting all in ages in good agreement based on 1*σ* uncertainties. We are thus confident that we have determined a robust *D*_e_, provided that anomalous fading is absent in the studied samples what is supported by the conducted fading test. These laboratory tests, however, cannot detect long-term fading which would underestimate the dose and the age.

By contrast, dose rate determination for the two investigated samples is more prone to systematic errors. The most significant factors for dose rate uncertainties comprise the estimated moisture content of the sedimentary matrix over the burial period, the overburden of pyroclastic surge deposits since the time of eruption, potential non-uniformity of the β- and γ-radiation fields as well as the original size distribution of quartz fragments in the sampled matrix.

The moisture content of bulk samples was estimated based on laboratory measurement of the saturation water content (~13 wt.%), and the assumed representative value taken for age calculations spans a range considered as covering a large part of physically meaningful values (10 ± 4 wt.%). The uncertainty of this factor is hence reflected in the overall error margin of TL ages.

While in many settings the cosmic dose rate contributes only a minor proportion of the total dose rate (based on dose rate generated by sediment of average crustal composition, e.g. loess [26]), it makes up ~10% of the total dose rate for the Nyos maar samples. An erroneous calculation of cosmic dose rate thus causes a relatively larger impact on the final age. Although we considered the best available information, a systematic error inherent in cosmic dose rate assessment cannot be ruled out.

Comparatively large granite rocks (cm-scale) can occasionally be found within the Nyos maar tuff. Although we carefully tried to consider only material for luminescence measurements that was located as far as possible away from such granite rocks, their potential influence on the actual dose rate and hence age must be discussed. Unfortunately, it was not possible to conduct *in-situ* measurements of the γ-radiation field directly at the sampling spot. Previous analyses of similar granitoids in the region showed that with approximate radioelement contents of K ~ 3 wt.%, Th ~ 20 μg g^-1^ and U ~ 2 μg g^-1^ these rocks generate a stronger γ-radiation field than the surrounding maar tuff (see Table 1). In case such a piece of granite was located in the vicinity (within ~ 30 cm) of the sampling spot in the tuff during burial, the effective dose rate would have been higher, with the consequence that ages become younger. In that sense, the presented ages (Table 2) should be regarded as maximum estimates, at least in view of the potential impact of spatial γ-radiation heterogeneities in the tuff.

Due to attenuation of external β-radiation within the quartz grains, their size distribution plays an important role for accurate dose rate calculation. However, no sorting of grains after the phreatomagmatic eruption took place prior to deposition (as is the case for wind-blown and water-lain sediments), and bedrock structure as well as the spatio-temporal dynamism of the eruption might have led to strongly varying conditions of rock fragmentation for different locations within the maar diatreme. Hence, the range of quartz grain sizes found in the Nyos maar tuff is comparatively large. Given that the attenuation of β-radiation emitted by K, Th and U isotopes follows an approximately inverse linear relationship with grain size up to ~ 2 mm [40,43], it is expected that averaging effects circumvent major systematic errors in dose rate assessment, once a representative average grain size of quartz clasts has been determined.

Finally, it has to be noted that the success of luminescence methods in accurately dating phreatomagmatic processes critically relies on the type of bedrock being ejected during the volcanic eruption and incorporated into the volcanic deposits. While our study produced promising results for shattered granitic country rock, gneisses might be associated with quartz luminescence properties less advantageous for dating purposes. In such cases, focusing on feldspar as the target mineral could represent an alternative approach, although allowing for anomalous fading will then require additional measurement and correction procedures [44]. Also, the effects of high-pressure shock waves on the luminescence properties of quartz as experienced during a phreatomagmatic explosion are still poorly studied. Despite the encouraging findings obtained for the Nyos maar samples, further methodological studies are mandatory to fully explore the potentials and pitfalls of this still experimental technique.

### Implications for hazard assessment

Despite all methodological challenges faced in dose rate assessment, we conclude that luminescence dating of phreatomagmatic eruptions in the CVL is a promising alternative to other dating techniques (e.g., ^14^C, K/Ar, U-series), which have produced ages for the Nyos maar differing by three orders of magnitudes [23]. It thus appears prudent to expand the application of luminescence methods to other sites of the CVL. Especially for Holocene and Late Pleistocene eruption sites too young for precise ^40^Ar/^39^Ar dates and lacking datable material for ^14^C analyses, luminescence dating is a valuable tool to complement and refine existing chronologies of active volcanism along the CVL. Such chronologies play a key role in risk evaluation of future eruptions [2,45].

Our new averaged maximum TL age estimate of 12.3 ± 1.5 ka is in rough agreement with the proposed eruption age of the Nyos maar between 5 and 10 ka ago (U-series dating [23]). The Nyos maar is the only one among the existing ~20 lake-bearing maars of the CVL, for which chronometric constraints are available, because of the aforementioned difficulties in determining ages for young volcanic activity. TL dating methods may thus also help to confine the age of the Monoun maar about 100 km south of the Nyos maar. A systematic dating of these maars can generate a better understanding of maar volcanic eruption cycles and possibly reveal indicative characteristics that may help classifying maar lakes of the CVL in terms of relative risk.

## Conclusion

The combination of various TL dating techniques applied to granitic quartz clasts in pyroclastic surge deposits of the Nyos maar allowed us to determine a direct maximum age estimate for the eruption of 12.3 ± 1.5 ka. Despite some methodological challenges related to dose rate calculation, luminescence methods represent a promising approach for completing the eruption chronologies not only of Holocene and Late Pleistocene volcanoes of the CVL, but in comparable settings worldwide.

## Supporting information

S1 Fig LM-OSL curve deconvolution of one aliquot of sample BT1611. The sample received a regenerative β-dose of 500 Gy and was preheated to 200°C for 10 s. Curve fitting was performed with the ‘fit_LMCurve’ function implemented in the R package ‘Luminescence’, version 0.6.4 [29–31]. A different number of individual components was adopted for the fitting procedures, and the lowest number of components above which no noticeable increase in the fitting quality parameter pseudo-R^2^ occurred is shown. The ratio of successive values of the calculated photoionisation cross-section indicate that components 1‒5 correspond to the fast, medium, s1, s2 and s3 component as described in [46] and [47]. Absolute values for the photoionisation cross-section were not calculated because the optical stimulation power density at sample position is not known well enough for that purpose. OSL decay curves recorded following regenerative β-doses of 100 Gy did not reduce to background level after 60 s of stimulation, indicating that the influence of non-fast-components at smaller doses is still high enough to render the OSL signal unsuitable for dating.
(PDF)

S2 Fig LM-OSL curve deconvolution of one aliquot of sample BT1612. Measurement and data evaluation procedures were the same as for sample BT1611 (S1 Fig).
(PDF)

S3 Fig Results of the fading test for one aliquot of sample BT1612. The plot shows the *L*_x_/*T*_x_ ratio (normalized to the *L*_x_/*T*_x_ value after zero pause) against the logarithm of the delay time t* between irradiation and measurement (again normalized to the delay after zero pause). The delay time t* consists of half of the irradiation time, the machine time needed to start the TL measurement after end of irradiation and a pause of variable duration [34].
(PDF)

S4 Fig Original size of quartz grains contained in the maar tuff. The blue grid has a mesh size of 1 mm.
(TIF)
