# Supplementary figures and images for: New thermoluminescence age estimates for the Nyos maar eruption (Cameroon Volcanic Line)
Source: PLoS One. 2017 May 30;12(5):e0178545. doi: 10.1371/journal.pone.0178545 (PMC5448780; doi:10.1371/journal.pone.0178545)

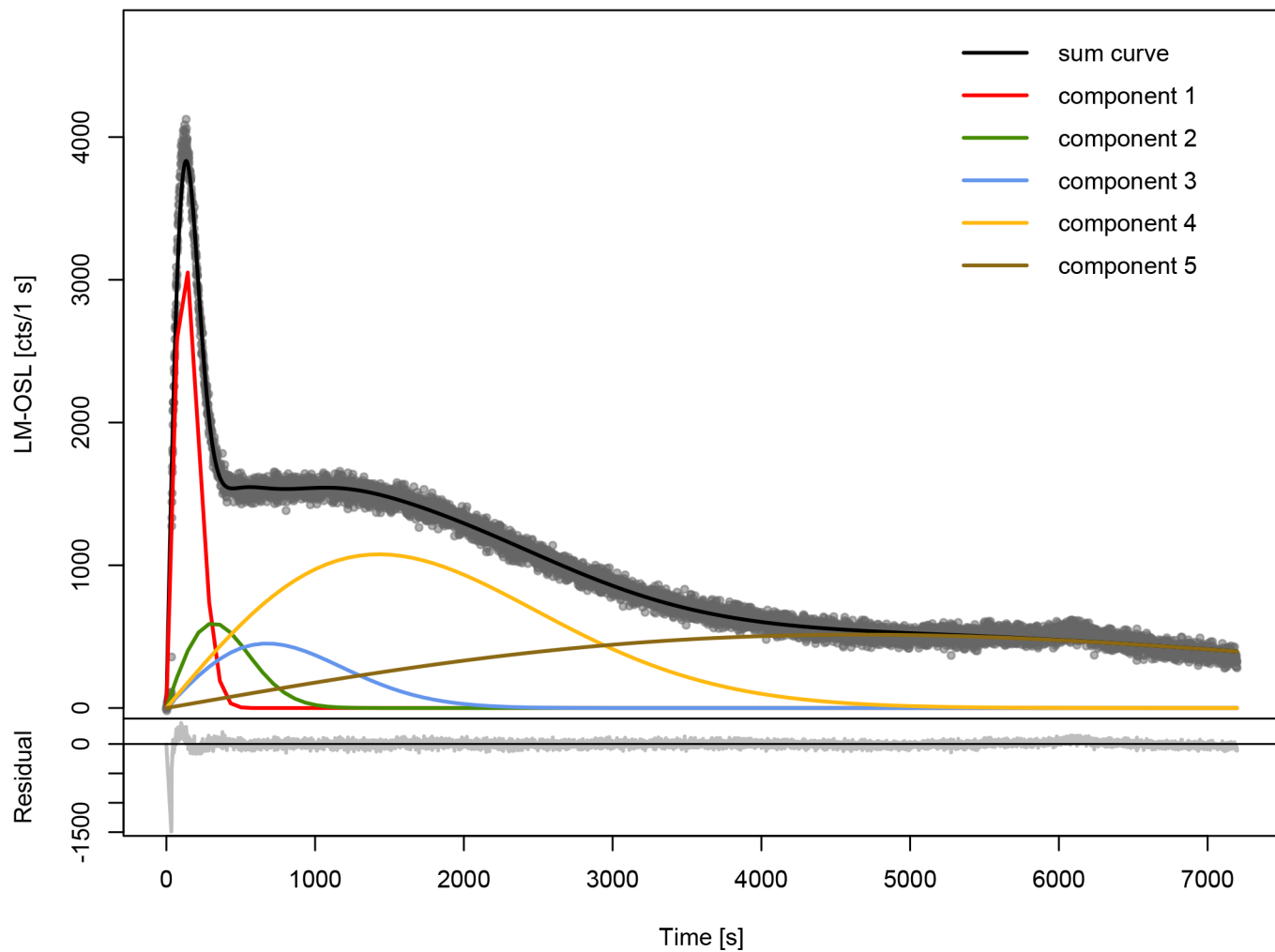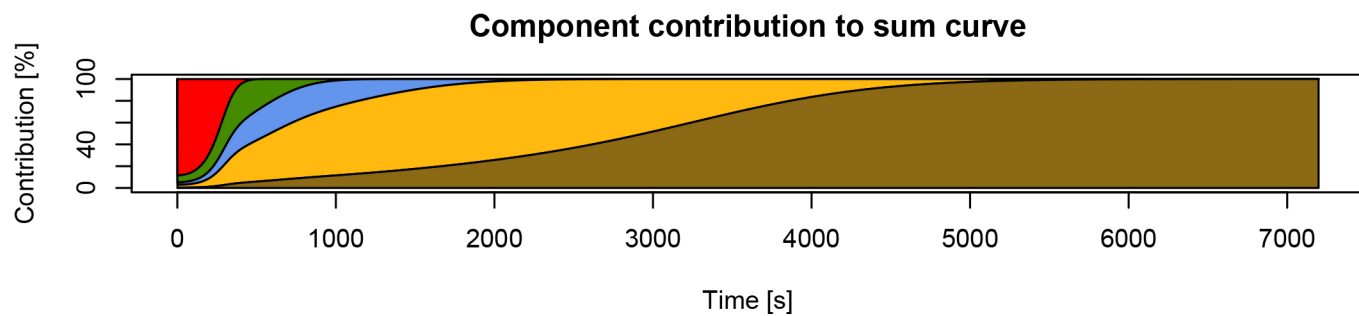

Supplement: S1 Fig — The sample received a regenerative β-dose of 500 Gy and was preheated to 200°C for 10 s. Curve fitting was performed with the ‘fit_LMCurve’ function implemented in the R package ‘Luminescence’, version 0.6.4 [29–31]. A different number of individual components was adopted for the fitting procedures, and the lowest number of components above which no noticeable increase in the fitting quality parameter pseudo-R2 occurred is shown. The ratio of successive values of the calculated photoionisation cross-section indicate that components 1‒5 correspond to the fast, medium, s1, s2 and s3 component as described in [46] and [47]. Absolute values for the photoionisation cross-section were not calculated because the optical stimulation power density at sample position is not known well enough for that purpose. OSL decay curves recorded following regenerative β-doses of 100 Gy did not reduce to background level after 60 s of stimulation, indicating that the influence of non-fast-components at smaller doses is still high enough to render the OSL signal unsuitable for dating. (PDF) [file pone.0178545.s001.pdf]

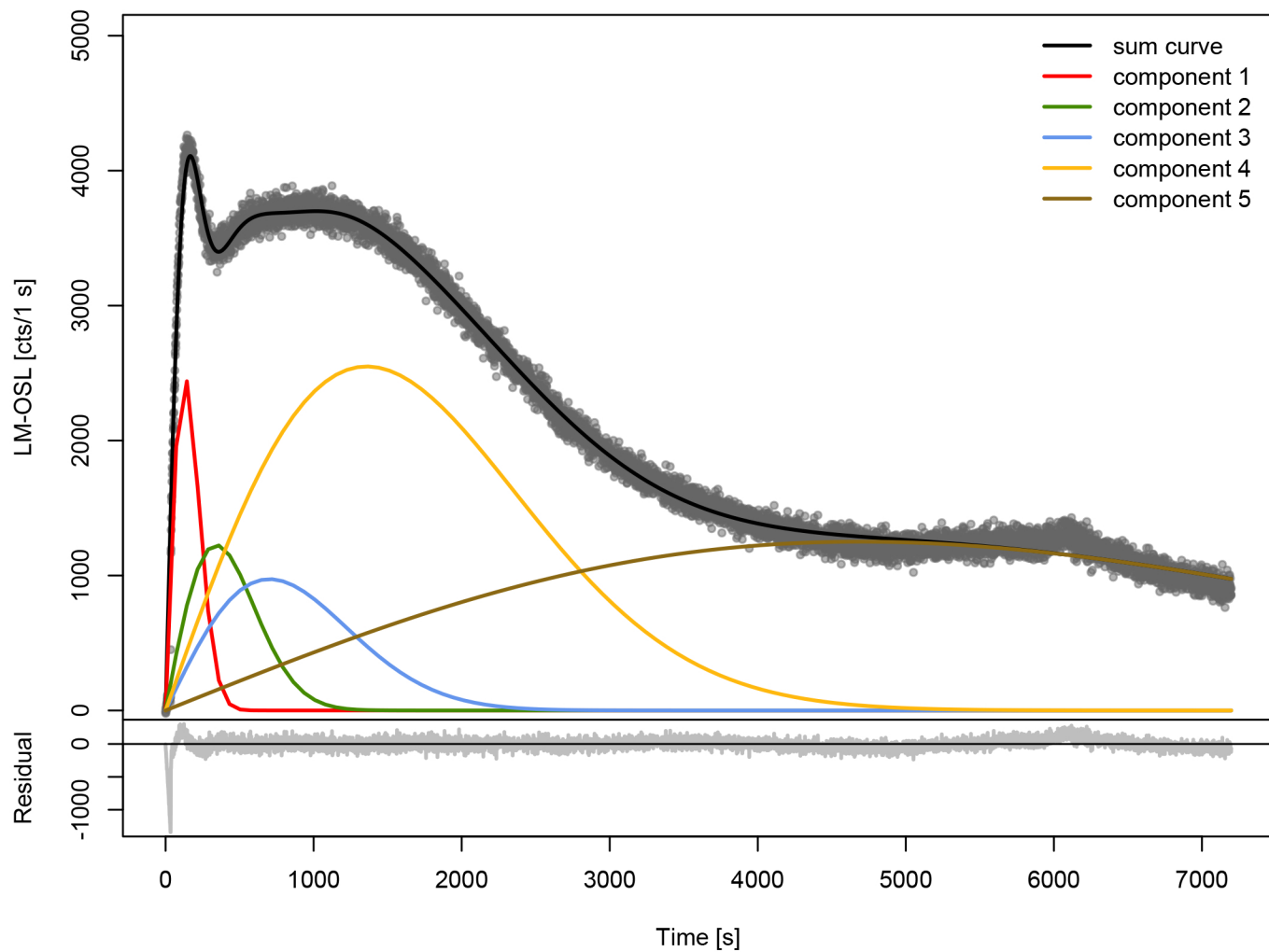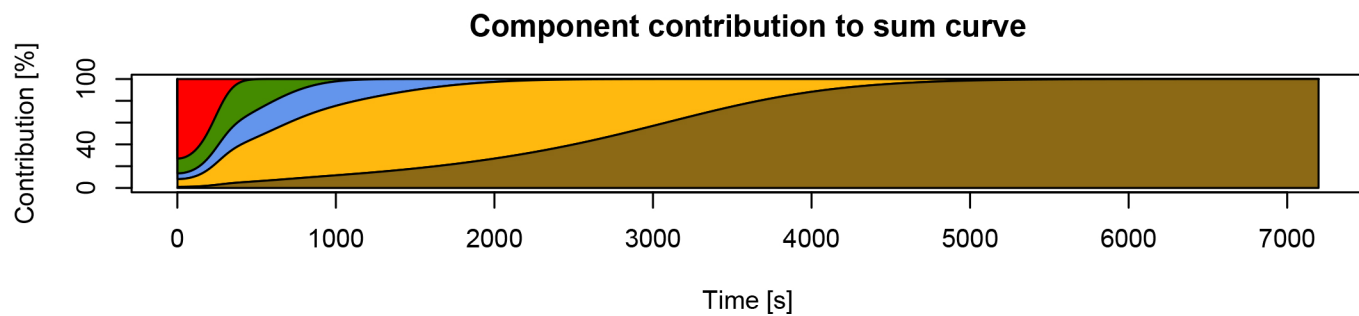

Supplement: S2 Fig — Measurement and data evaluation procedures were the same as for sample BT1611 (S1 Fig). (PDF) [file pone.0178545.s002.pdf]

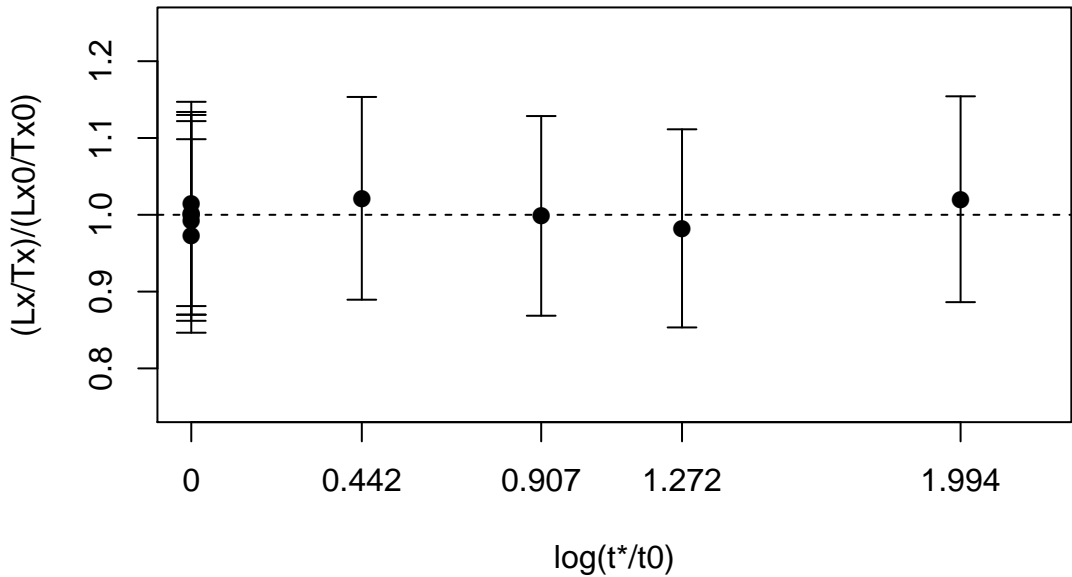

Supplement: S3 Fig — The plot shows the Lx/Tx ratio (normalized to the Lx/Tx value after zero pause) against the logarithm of the delay time t* between irradiation and measurement (again normalized to the delay after zero pause). The delay time t* consists of half of the irradiation time, the machine time needed to start the TL measurement after end of irradiation and a pause of variable duration [34]. (PDF) [file pone.0178545.s003.pdf]

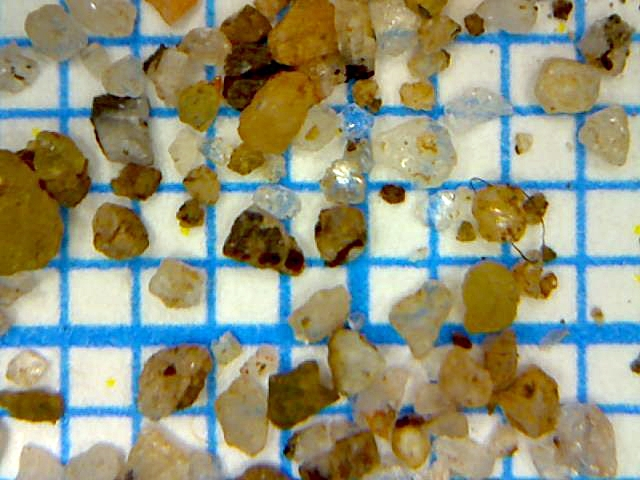

Supplement: S4 Fig — The blue grid has a mesh size of 1 mm. (TIF) [file pone.0178545.s004.tif]
